# Human Decision-making in an Artificial Intelligence–Driven Future in Health: Protocol for Comparative Analysis and Simulation

**DOI:** 10.2196/42353

**Published:** 2022-12-23

**Authors:** Nandini Doreswamy, Louise Horstmanshof

**Affiliations:** 1 National Coalition of Independent Scholars Dickson, ACT Australia; 2 Faculty of Health Southern Cross University Lismore, New South Wales Australia

**Keywords:** human decision-making, AI decision-making, human-AI interaction, human roles, artificial intelligence, nonclinical health services, health policy, health regulation

## Abstract

**Background:**

Health care can broadly be divided into two domains: clinical health services and complex health services (ie, nonclinical health services, eg, health policy and health regulation). Artificial intelligence (AI) is transforming both of these areas. Currently, humans are leaders, managers, and decision makers in complex health services. However, with the rise of AI, the time has come to ask whether humans will continue to have meaningful decision-making roles in this domain. Further, rationality has long dominated this space. What role will intuition play?

**Objective:**

The aim is to establish a protocol of protocols to be used in the proposed research, which aims to explore whether humans will continue in meaningful decision-making roles in complex health services in an AI-driven future.

**Methods:**

This paper describes a set of protocols for the proposed research, which is designed as a 4-step project across two phases. This paper describes the protocols for each step. The first step is a scoping review to identify and map human attributes that influence decision-making in complex health services. The research question focuses on the attributes that influence human decision-making in this context as reported in the literature. The second step is a scoping review to identify and map AI attributes that influence decision-making in complex health services. The research question focuses on attributes that influence AI decision-making in this context as reported in the literature. The third step is a comparative analysis: a narrative comparison followed by a mathematical comparison of the two sets of attributes—human and AI. This analysis will investigate whether humans have one or more unique attributes that could influence decision-making for the better. The fourth step is a simulation of a nonclinical environment in health regulation and policy into which virtual human and AI decision makers (agents) are introduced. The virtual human and AI will be based on the human and AI attributes identified in the scoping reviews. The simulation will explore, observe, and document how humans interact with AI, and whether humans are likely to compete, cooperate, or converge with AI.

**Results:**

The results will be presented in tabular form, visually intuitive formats, and—in the case of the simulation—multimedia formats.

**Conclusions:**

This paper provides a road map for the proposed research. It also provides an example of a protocol of protocols for methods used in complex health research. While there are established guidelines for a priori protocols for scoping reviews, there is a paucity of guidance on establishing a protocol of protocols. This paper takes the first step toward building a scaffolding for future guidelines in this regard.

**International Registered Report Identifier (IRRID):**

PRR1-10.2196/42353

## Introduction

### Background

Nonclinical health services such as health regulation and health policy are more extensive and complex than clinical health services in their scope and scale. They can be viewed regionally, nationally, or globally. Furthermore, health regulation and health policy often intersect and overlap. For example, during the COVID-19 pandemic, health regulation and health policy provide a continuum of rules, laws, and public health measures that may vary from one region to another and from country to country. An array of organizations at different levels of government may be involved in the oversight and control of health regulation and health policy, with input and influence from numerous private entities and commercial concerns. Therefore, there are often differences in perspective and tensions between opposing interests. For all these reasons, health regulation and health policy can be viewed as “complex health services.” Health care, then, can be broadly divided into clinical health services and complex health services.

Artificial intelligence (AI) is beginning to transform complex health services. It can recognize patterns and compute correlations far beyond human capacity [1]. For instance, machine learning can be applied to big data at the population level from electronic health records, medical imaging, and genomic data [2] to predict the incidence of disease in a population. AI is used to analyze data from numerous digital resources and monitor social media to assist with critical public health initiatives such as the timely supply of vaccines [3]. AI analysis of social media has shed light on important issues such as cigarette smoking [4], unlawful sales of opioids online [5], and the thinking that underlies vaccine hesitancy [3].

However, AI-driven health policy and health regulation may not be as accountable, unbiased, or transparent as required in health care and may be prone to incorrect or unfair decisions [6]. AI can entrench existing biases or introduce other forms of bias in decision-making [7]. AI is an “anormative black box” [8]—it is possible to know its inputs and outputs but not its internal reasoning or logic. Furthermore, its algorithms are often exceedingly long, complex, and essentially disconnected from sense-making, making it a challenge to criticize or audit AI systems [8]. Importantly, there are legislative and regulatory gaps in the policies and ethics that should govern AI such as bias, lack of transparency in AI algorithms, privacy and data governance concerns, and cybersecurity issues [9]. Appropriate safety policies and precautions, risk management matrices, and areas of responsibility still need to be developed to address these concerns [10].

Regardless, AI is taking a prominent role in decision-making and is being used to solve increasingly complex tasks [11]. Early forms of AI such as machine learning and decision support systems are becoming increasingly important in decision-making in complex health services. These forms of AI collate, filter, search, and find patterns in big data, enabling human decision makers to make evidence-based decisions at speed [12]. In most nations and jurisdictions, AI is not currently allowed to make the final decisions in health policy and health regulation [13]. However, its footprint in decision-making is growing steadily. While humans are leaders, managers, and decision makers in complex health services today, it is unclear whether they will continue to have meaningful decision-making roles in an AI-driven future.

Complex health services are beginning to incorporate several advanced AI techniques, such as deep learning and natural language processing [14], into sophisticated AI-based decision support systems [2]. It is only a matter of time before AI begins to drive or dominate complex health services. Therefore, this research is timely and essential.

### Research Design

The proposed research is designed as a four-step project, divided into two phases. Phase 1 aims to address the question of whether humans will continue to have meaningful decision-making roles in complex health services in an AI-driven future, based on any unique human attributes that may influence decision-making for the better. This phase consists of three distinct steps. The first step is a scoping review of literature to identify and map attributes that influence human decision-making in complex health services. The second step is a scoping review of literature to identify and map the attributes that drive AI decision-making in complex health services. The third step aims to provide a comparative analysis of the decision-making attributes of humans and AI, and make clear recommendations for future research in this area. It may include a narrative comparison, followed by a mathematical comparison, of these two sets of attributes.

Phase 2 aims to explore the question of whether humans will compete, cooperate, or converge with AI to continue in decision-making roles. This phase consists of a simulation, which is the fourth and final step of the proposed research. The simulation is based on mathematical modeling, where human and AI attributes are used to create virtual *agents* in an environment that closely replicate complex health services.

### Significance and Expected Outcomes of This Research

There is an urgent need to determine whether humans are likely to continue in meaningful decision-making roles in complex health services in an AI-driven future. There is a dearth of literature on the role that AI may play in decision-making in this context. More broadly, this research is expected to contribute to addressing the question of whether humans will continue to play a meaningful role in a future likely to be dominated by AI [15]. The increasing sophistication of algorithms, matched by advances in data acquisition and data storage, is integrating AI into many facets of life [16]. This presents both opportunities and challenges. Therefore, while harnessing AI’s potential, it is important to develop strategic frameworks that identify and balance benefits and risks early.

## Methods

### Protocol for the Scoping Reviews of the Literature

This is the protocol for the first two steps of phase 1:

1. A scoping review of the literature to identify and map attributes that influence human decision-making in complex health services
2. A scoping review of the literature to identify and map the attributes that influence AI decision-making in complex health services

#### Method

Both scoping reviews are based on the framework recommended by Peters et al [17]. The framework is based on PCC (Population, Concept, and Context), which has been adapted for each scoping review. In keeping with the framework, the scoping reviews focus on the headings set out below.

##### Titles and Review Questions

The title of the first scoping review is “Attributes That Influence Human Decision-making in Complex Health Services: A Scoping Review.” The research question is what attributes have been reported in the literature that influence human decision-making in complex health services?

The title of the second scoping review is “Attributes that Influence AI Decision-Making in Complex Health Services: A Scoping Review.” The research question is what attributes have been reported in the literature that influence AI decision-making in complex health services?

##### Inclusion and Exclusion Criteria

The reviews will consider all articles relating to human decision-making and AI decision-making in complex health services. The populations of interest are human decision makers and AI decision makers. The concept is decision-making in the context of complex health services.

Articles that focus on decision-making in areas not relevant to the research questions will be excluded. For example, articles focusing on the following topics will be excluded: clinical health; maternal health, abortion, and discrimination against women; decision space for health recruitment; legal matters; environmental health, contamination, and toxicity; computers, human-computer interaction, and automated decision rules; mathematical modeling; and assessment of organizational performance.

##### Types of Evidence Sources

The reviews will consider a wide range of evidence sources, including empirical research (eg, qualitative and quantitative studies), case studies, expert opinions, critiques, commentaries, editorials, textual data, and narrative data. However, to ensure that these sources are of a reasonable quality, the reviews will include peer-reviewed journal articles only, and exclude book chapters, conference papers, and gray literature.

##### Search Strategy

An initial informal exploration will be undertaken to determine optimal search system and database combinations. Suitable search systems thus identified will be searched for peer-reviewed literature. The search will include all available databases in these search systems. The search terms used will be as logical, relevant, and comprehensive as possible.

##### Evidence Screening and Selection

**Figure 1:**
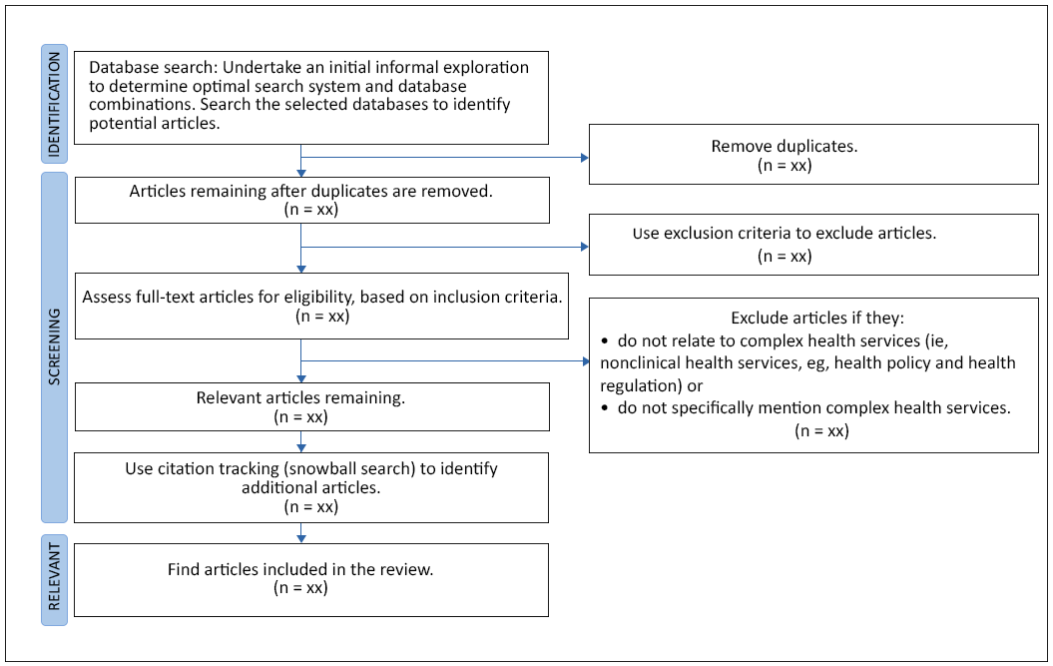
Flow diagram based on the PRISMA-ScR (Preferred Reporting Items for Systematic Reviews and Meta-Analyses Extension for Scoping Reviews) [18].

The search will be limited to peer-reviewed journal articles in English only because of constraints on budget and time. However, no limits will be placed on the year of publication to try and capture articles through time that may reference seminal works on decision-making in the context of complex health services. Article screening and selection will be based on PRISMA-ScR (Preferred Reporting Items for Systematic Reviews and Meta-Analyses Extension for Scoping Reviews) [18] (Figure 1).

##### Data Extraction

A framework was developed for the selection, data extraction, and categorization of articles based on the work of Sav et al [19] and used as a standardized process to extract data. The process includes extracting the first author, year of publication, title, country of the first author, language of publication, source (search system), article type, and summary of the topic of the article.

##### Data Analysis

Data analysis will be undertaken to identify attributes mentioned in the literature reviewed, conduct a frequency count of attributes (analyze how many articles mention a given attribute), and identify broad qualitative themes.

##### Presentation of Results

Based on the framework for data analysis, the results will be presented in tabular form and in visual diagrammatic formats such as tree maps.

#### Discussion: Scoping Reviews

Each scoping review will conclude with a discussion of the salient findings.

### Protocol for the Comparative Analysis

The third step of phase 1 is a comparative analysis of human and AI attributes. It is a narrative comparison, followed by a mathematical comparison, of two sets of attributes—human and AI. This analysis will investigate whether humans have one or more unique attributes that could influence decision-making for the better and ensure that humans continue in meaningful decision-making roles in complex health services.

There is a growing awareness that appropriate methods are required to address the increasing complexity of health research. Therefore, the narrative comparison may not only include frames of reference, logical arguments, and links to each point in the argument but also incorporate the hermeneutic spiral. This is the iterative process of comparative analysis that “moves back and forth between individual elements of the text and the whole text in many cycles” [20].

For the mathematical comparison, qualitative comparative analysis (QCA) may be appropriate, as it is an established method used in social science research [21,22] and public health research [23]. It can be applied to the complexity of the proposed research, provide the in-depth analysis required, and produce broad enough contexts for generalizations to be made. Furthermore, as it is based on set theory [24], it can be used to frame human and AI attributes as sets and examine any relationships between these sets. The data sets generated in the proposed research are likely to be of an appropriate size for QCA to be applied. However, if the size is not suitable for QCA, related methods of analysis may be used. For example, cross-case analysis [21] could be used for small data sets and linear regression analysis [22] for large data sets.

#### Method

A comparative analysis will be performed on the human and AI attributes identified and mapped in the first and second scoping reviews of phase 1. The mathematical comparison will proceed as follows:

- Each set of attributes (human and AI) will be viewed as a mathematical set.
- Each set could be divided into subsets such as unique and nonunique attributes.
- A comparative analysis of these sets and subsets will then be undertaken to determine whether humans may have one or more unique attributes that influence decision-making.

##### Tools

Software suited for QCA will be used to complete this step. For instance, software such as NVivo (QSR International), ATLAS.ti (ATLAS.ti Scientific Software Development GmbH), Quirkos (Quirkos Software), or Tosmana (University of Trier) may be suitable.

#### Discussion: Comparative Analysis

The comparative analysis will conclude with a discussion of the salient findings.

### Protocol for the Simulation

The fourth and final step in phase 2 of the proposed research is a simulation based on mathematical modeling. Its purpose is to explore whether humans are likely to compete, cooperate, or converge with AI to continue in meaningful decision-making roles in complex health services. This will be achieved by creating a virtual system, using mathematical modeling, that closely resembles the nonclinical health care environment. Human attributes identified in the first scoping review in phase 1 will be used to simulate a human decision maker within the simulated environment. Similarly, AI attributes identified in the second scoping review will be used to simulate an AI decision maker. Simulations will then be conducted to explore, observe, and document whether humans are likely to compete, cooperate, or converge with AI.

Simulations based on mathematical modeling inform decisions in many health care settings [23]. In the last 5 to 6 years, three contemporary models have been successfully used in simulations in health care design and prediction: the system dynamics model (SDM), the agent-based model (ABM), and the hybrid SDM-ABM model. One or more of these models could be deployed in the simulation in phase 2 of the proposed research. These models use the concept of players, known as agents, who interact in a system or environment.

SDM can be used to simulate changes to a system over a period of time [25]. It provides an effective view of the system, or environment, at the macro level. ABM is effective in simulating environments and interactions between one or more decision-making agents [26]. These agents can make decisions based on their own attributes, interactions with other agents, interactions with the modeled environment, or a combination of these [27]. ABM provides effective views of agents and environments at the micro level. The hybrid SDM-ABM model provides both macro and micro views of environments and agents [28,29].

#### Method

The simulation may use the SDM, ABM, or hybrid model, or the most appropriate combination of the three.

##### Tools

Software tools will be required to complete the simulation. Maple 2021 (Maplesoft) software is currently the most suitable, as it has the depth and breadth needed for academic research that involves the simulation of complex, dynamic systems. This software has been used for complex simulations in fields as diverse as finance [30] and robotics [31,32].

#### Discussion: Simulation

The simulation will conclude with a discussion of the salient findings.

### Ethical Considerations

Ethics approval is not required because this research project involves scoping reviews of literature, mathematical models, and simulation. It does not include studies that involve humans or other living beings.

## Results

The results will be presented in tabular form and visually intuitive formats. For the comparative analysis and simulation, results will be presented in digital storytelling and multimedia formats as well. Table 1 shows the protocol for the presentation of results.

**Table 1:** Protocol for the presentation of results.

Step (phase)	Study	Tabular formats?	Heat maps?	Digital storytelling?	Multimedia?
Step 1 (phase 1)	Scoping review to identify and map human attributes that influence decision-making in complex health services	✓	✓		
Step 2 (phase 1)	Scoping review to identify and map AI^a^ attributes that influence decision-making in complex health services	✓	✓		
Step 3 (phase 1)	Comparative analysis of the two sets of attributes: human and AI	✓	✓	✓	✓
Step 4 (phase 2)	Simulation of a health regulation and policy environment with human and AI agents	✓	✓	✓	✓

^a^AI: artificial intelligence.

## Discussion

There are established guidelines for a priori protocols [33] that are developed before undertaking scoping reviews in health research. Numerous examples of such protocols are found in the literature. However, there is a dearth of guidance on establishing a protocol of protocols for methods used in complex health research. This paper takes the first step toward building a scaffolding for future guidance in this regard. It provides not only a roadmap for the proposed research but also an example of a protocol of protocols. This may be relevant and useful in spheres of complex research such as human-AI interaction and health informatics. This may also be an opportunity to further investigate the issue of bias, the dominance of rationality, and the likely influence of intuition.

## Abbreviations

ABM: agent-based model
AI: artificial intelligence
PCC: Population, Concept, and Context
PRISMA-ScR: Preferred Reporting Items for Systematic Reviews and Meta-Analyses Extension for Scoping Reviews
QCA: qualitative comparative analysis
SDM: system dynamics model
